# Transcranial magnetic stimulation in cocaine use disorder and the risk of seizure: a review of the evidence

**DOI:** 10.3389/fpsyt.2026.1748184

**Published:** 2026-02-04

**Authors:** Caesar G. Imperio, Eric Parmon, Vaughn R. Steele, Derek Blevins, Rebecca Chalme, Jonathan M. Wai, Kathleen Brady, Markus Heilig, Colleen A. Hanlon, Frances R. Levin, Diana Martinez

**Affiliations:** 1 New York State Psychiatric Institute, Division on Substance Use Disorders, New York, NY, United States; 2 Columbia University Irving Medical Center, Department of Psychiatry, New York, NY, United States; 3 Department of Psychiatry, Yale School of Medicine, New Haven, CT, United States; 4 Institute of Living, Hartford Hospital/HealthCare, Olin Neuropsychiatry Research Center, Hartford, CT, United States; 5 Center for Brain & Mind Health, Yale University, New Haven, CT, United States; 6 Department of Psychiatry and Behavioral Sciences, Medical University of South Carolina, Charleston, SC, United States; 7 Department of Psychiatry, Center for Social and Affective Neuroscience, Linköping University, Linköping, Sweden; 8 Department of Cancer Biology, Wake Forest University School of Medicine, Winston-Salem, NC, United States; 9 Department of Medical Affairs, BrainsWay, Winston-Salem, NC, United States

**Keywords:** addiction, cocaine, intoxication, seizure, toxicity, transcranial stimulation

## Abstract

Transcranial magnetic stimulation (TMS) has emerged as a promising intervention for cocaine use disorder (CUD). However, a key concern when employing TMS in CUD is the potential risk of seizures. Our goal was to assess seizure risk in individuals with CUD undergoing TMS and to propose parameters that could mitigate it. Our review of the literature indicated that seizures are primarily associated with high-dose cocaine use necessitating urgent medical care – and that the risk is likely low outside of this setting. Thus, to mitigate potential seizure risks during TMS sessions, we suggest an assessment of recent cocaine use and an evaluation for cocaine toxicity. Additionally, rechecking motor threshold levels during treatment with TMS is recommended, especially if patterns of cocaine use change. Previous studies of TMS in CUD reported on two seizures that were linked to recent cocaine use rather than proximity to TMS treatment itself. Future research should document the timing of cocaine use relative to TMS sessions to further ensure the safety of this therapeutic approach.

## Introduction

Cocaine use disorder (CUD) can lead to impaired social and vocational functioning, lost opportunities, and the increased risk of morbidity and mortality (1, 2). However, effective pharmacologic treatment remains elusive (3, 4). Clinical trials have searched for medications that could help patients reach remission, but no clear options have emerged. While studies suggest that some pharmacotherapy (such as long-acting stimulants or combined medications) may help some patients, there remains a need for therapeutic options (1, 2, 5).

Research shows that transcranial magnetic stimulation (TMS) may serve as a treatment for CUD by reducing craving and cocaine use (6, 7). Although a pivotal trial has yet to be published, early studies using heterogeneous approaches – including imaging, open-label trials, retrospective record reviews, and randomized controlled trials – have shown promise for this refractory disorder.

Seizure is a rare but concerning potential adverse event associated with TMS (8, 9). While the incidence is low, there is a concern of increased seizure risk among individuals with CUD based on data showing that cocaine exposure itself can cause a seizure (10–26).

Given the promise of TMS for CUD, our goal was to characterize the risk of seizure and to identify methods to reduce it. We began with a review of seizures and cocaine use, followed by an analysis of studies where cocaine was safely administered in laboratories. We then reviewed clinical trials of TMS in CUD, with a focus on reports of seizures in this population. From this analysis, we provide suggestions that can be used to mitigate the seizure risk on TMS in CUD.

## Cocaine use and the risk of seizure

In the 1980s, cocaine production and consumption increased across the globe. Clinical researchers soon identified the association between cocaine exposure and seizure, which is now a recognized potential adverse event. We reviewed the literature on this topic, starting with a systematic review by Sordo et al. (27), which identified 11 cross-sectional studies (10–20) and one case-control study (21) reporting on seizure prevalence among individuals using cocaine. We then replicated the search of Sordo et al., to identify additional studies published since their 2013 review by searching Medline, EMBASE, and PsycINFO using the terms “cocaine, cocaine-related disorders, cocaethylene, and seizures” and excluded unverified reports of seizure.

This returned a total of 17 studies (**Table 1**), which show that seizure was seen in 1% to 27% of individuals who had used cocaine recently (intravenous, smoked, insufflated, or oral) (10–26). As shown in **Table 1**, all studies included patients receiving acute medical care in emergency departments or hospitals due to cocaine use alone or due to illness where cocaine use was a contributing factor. The highest prevalence (27%) was reported in a retrospective analysis of cocaine-related deaths, suggesting that the greatest risk of seizure may be during a fatal overdose (23).

**Table 1:** Studies reporting on the presence of seizure in participants using cocaine, in chronological order.

Study	Participant population and reported seizure
Case-control study	
Ng et al., 1990 (21)	Cases: hospitalized patients with first episode non-febrile seizure (n=308); Controls: hospitalized patients without seizure (n=294). Subjects interviewed about drug use (cocaine, amphetamines, cannabis, LSD, PCP, heroin, methadone, methaqualone). AOR = 1.05 for any cocaine use; AOR = 1.69 for cocaine use within 1 day of admission
Retrospective record reviews of hospital charts	
Lowenstein et al., 1987 (10)	ED visits + hospital admissions of patients with cocaine use as the primary problem (n=1275). Seizure reported in 2.3% of patients.
Choy-Kong et al., 1989 (11)	Hospital admissions of patients where diagnosis cocaine ‘use or abuse’ in record (n=283). Seizure reported in 2.8%; clear temporal association seen in 1.4%.
Derlet et al., 1989 (12)	ED visits of patients presenting with drug use or overdose (n=137 attributed to cocaine). Seizure reported in 8.8% of cocaine exposed.
Pascual-Leone et al., 1990 (13)	ED visits of patients with medical complications caused by cocaine intoxication (n=474). Seizure reported in 16.9% among patients with a history of seizure; among patients with no seizure history, rate was 7.9%
Brody et al., 1990 (14)	ED visits of patients using cocaine, including use with other drugs (n=216). Seizure reported in 4.3%.
Dhuna et al., 1991 (15)	Hospital admissions for non-traumatic complications of cocaine use (obstetric complications excluded) (n=945). Seizure reported in 10.4% (18.4% women, 6.2% men).
Rich et al., 1991 (16)	ED visits where “cocaine” was reported by patients within 72 hours or detected on toxicologic screen (n=144). Seizure reported in 4.2%.
Anta et al., 1998 (17)	ED visits directly related to patients’ cocaine use (injuries or obstetric/gynecologic, pediatrics excluded) (n=233). Seizure reported in 5.3%.
Blaho et al., 2000 (18)	ED visits with suspected or confirmed cocaine use, independent of chief complaint (n=111). Seizure reported in 3%.
Sanjurjo et al., 2006 (19)	ED visits where cocaine use occurred within “the previous hours” by patients (n=745). Seizure reported in 1.9%. Data from Spain.
Sopena et al., 2008 (20)	Patients hospitalized due to complications of cocaine use (n=177). Seizure reported in 6.2%. Data from Spain.
Bodmer et al., 2014 (24)	ED visits due to acute cocaine-related medical problems (n=165). Seizure reported in 4% of cocaine-only exposed; 1% with use of additional substances (mostly alcohol, tobacco, cannabis). Data from Switzerland.
Miro et al., 2019 (25)	ED visits involving cocaine use (n= 3002 total cases; n=2600 for powder + n=376 for smoked cocaine). Seizure reported in 4.5% in the powder cocaine and 5% in the smoked cocaine. Data from 14 European countries.
Glidden et al., 2022 (22)	Hospitalized patients receiving medical toxicology consultation (n=380 cocaine only; n=343 cocaine + opioid). Seizure reported in 8.1% of cocaine-only; 0% of cocaine and opioids.
Darke et al., 2023 (23)	Cocaine-related deaths from 2000-2021 (n=26 for cocaine only related deaths with witness observation). Seizure reported in 26.9% fatal cocaine only toxicity. Data from Australia.
Miro et al., 2025 (26)	ED visits for acute cocaine toxicity where cocaine was the only drug used except for alcohol (n=9365). Seizure reported in 4.8%. Data from 33 countries.

AOR, adjusted odds ratio; ED, emergency department; LSD, lysergic acid diethylamide; PCP, phencyclidine.

We then reviewed studies that included self-reports of seizure by cocaine users, which was not observed by medical personnel (28–32). The prevalence rates varied from 0.9% to 18.1%. This large range can be partly attributed to seizures being described as “fits, convulsions, fainting, and loss of consciousness” – such that these reports included non-seizure events. Nonetheless, these studies also indicated that seizures occurred in the setting of recent, high dose cocaine use that caused symptoms of physical and/or mental distress. Thius, this finding is similar to the studies with confirmed seizures witnessed by medical personnel.

Taken together, this research indicates that individuals who use cocaine and consequently require acute medical attention are at risk of seizure. We found no reports of increased seizure in persons with CUD outside of this setting. While the lack of data cannot provide definitive evidence, it is remarkable that there are no reports of increased rates of seizure associated with CUD itself.

With respect to TMS, this data suggests that seizure risk would be elevated following recent cocaine use, especially at toxic doses that induce illness – and could be low outside of this setting. In the next sections, we review methods that can be used to assess recent use and toxic doses.

## Evaluating recent cocaine use

A urine drug screen (UDS) is a reliable method for determining recent cocaine use. However, it detects benzoylecgonine (an inactive metabolite), which can persist in the urine for 2–4 days, well after cocaine has cleared the plasma (33). Additionally, although uncommon, a UDS can return a false negative result where cocaine is in the blood but benzoylecgonine has yet to appear in urine (34, 35). This can occur when cocaine use is very recent (within about 2 hours) before the metabolite is detectable (35, 36).

We sought to identify clinical parameters that could assess recent use – instead of a UDS or in addition to this test. For this goal, we reviewed studies where cocaine was taken by participants in a laboratory setting under close monitoring. This provided information on the signs and symptoms of recent cocaine use, which correlate with its pharmacokinetic profile.

The half-life of cocaine in the plasma is 45 to 90 minutes following smoked, intranasal, or intravenous administration (37–40). Once five half-lives have passed (or about 7.5 hours), plasma levels become undetectable (37–40). The subjective effects of cocaine intoxication include feeling high, alert, self-confident, social, talkative, anxious, irritable, and ‘on edge’ (41–46). These symptoms peak within about 5 minutes and resolve within about 30–45 minutes following a single dose (40, 45, 47–50). Repeated dosing extends the subjective effects of cocaine longer, beyond 100 minutes or more (41, 51, 52).

Increases in vital signs are another sign of cocaine intoxication. Studies show that cocaine increases heart rate by about 20–40 beats per minute and increases blood pressure (systolic and diastolic) by about 10–30 mmHg over baseline (41–46). These remain elevated for 2 to 3 hours following smoked or intravenous cocaine use (41, 44, 53).

Thus, the signs and symptoms of cocaine intoxication can be used to evaluate individuals for recent use, including subjective effects and vital signs. However, an additional approach is to establish a collaborative, non-judgmental dialogue with individuals about their patterns of cocaine use This could include the estimated dosage, timing, and route of administration – as well as a discussion of the potential risk of TMS in the setting of recent cocaine exposure.

## Assessing toxic cocaine use

The difference between a euphorigenic dose of cocaine (which produces a desired effect) and a toxic dose (which causes distress) can be miscalculated, even among individuals with regular cocaine use. A toxic dose, also called an overdose, leads to symptoms of physical and mental distress and requires a medical assessment. Thus, in addition to recent use, evaluating this parameter would improve safety.

The signs and symptoms of cocaine toxicity have been divided into three stages of increasing severity (54). Stage 1 includes central nervous system effects (headache, nausea, mydriasis, vertigo, twitching, pseudo-hallucinations, and pre-convulsive movements); changes in vital signs (hypertension, tachycardia, tachypnea, hyperthermia, ectopic beats); and psychiatric symptoms (paranoia, euphoria, confusion, aggression, agitation, emotional lability, restlessness). Stage 2 consists of additional symptoms of greater severity: encephalopathy, seizures, increased deep tendon reflexes, incontinence, arrhythmias, peripheral cyanosis, gasping, apnea, and irregular breathing (54). Stage 3 includes symptoms that can be life-threatening: areflexia, coma, fixed and dilated pupils, loss of vital function, hypotension, ventricular fibrillation, cardiac arrest, respiratory failure, cyanosis, and agonal breathing (54).

In reviewing the symptoms of cocaine toxicity, it is key to recognize that some individuals will experience stage 1 symptoms without progressing to stage 2 or 3 – and that not all symptoms listed above are required to meet criteria for cocaine overdose. Furthermore, some individuals might not have sought medical care despite having severe symptoms.

With respect to TMS, an evaluation of cocaine overdose should include both recent and past episodes. A recent episode could indicate that a clinical evaluation is needed to rule out ongoing illness, such as neurological or cardiac events. A past event may not be exclusionary for TMS, but should be assessed, since an overdose could have included neurological events, like ischemic stroke and intracerebral or subarachnoid hemorrhage (55, 56).

## TMS studies on CUD

**Figure 1:**
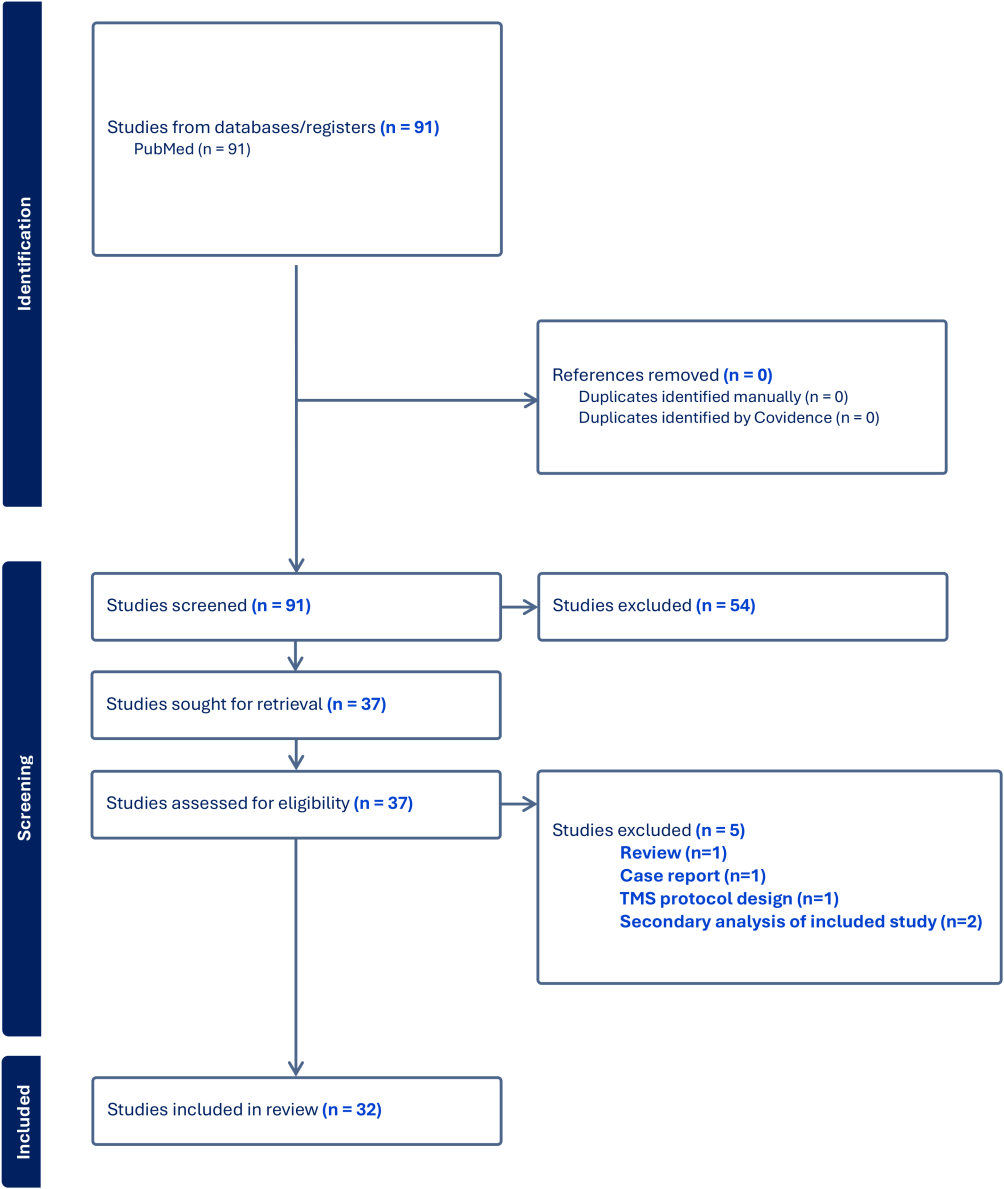
PRISMA flow diagram of study selection. PRISMA diagram detailing database search, number of abstracts screened, full texts retrieved, and number of studies included.

We reviewed studies of TMS in CUD, in order to evaluate two parameters: 1) whether an assessment of recent use was included prior to the delivery of TMS; and 2) whether seizure was reported. In May 2025, we searched PubMed using the term “cocaine and transcranial magnetic stimulation,” which returned 91 studies (**Figure 1**). Inclusion criteria consisted of investigational studies examining the use of TMS for CUD that were published in English. Publications were excluded if they were literature reviews, conference abstracts, commentaries, secondary analysis of already included data, or non-human investigations, leaving 32 studies for further review.

Of these, 11 studies required abstinence or a urine drug screen negative for cocaine prior to the delivery of TMS sessions (57–67). Notably, these studies used outcomes that were not affected by the requirement of abstinence, such as imaging, measures of craving, or cocaine self-administration. There were no reports of seizures.

We then reviewed publications that did not specifically require abstinence, in order to determine whether recent cocaine use was assessed prior to the delivery of TMS. This resulted in 21 studies (**Table 2**). As shown in **Table 2**, these publications assessed cocaine use with heterogeneous methods, including UDS, hair analysis, self-report, and reports from significant others (68–88). However, only five (of the 21) studies clearly reported that recent use was evaluated prior to the delivery of TMS (70, 74, 75, 81, 85). The remaining studies did not specify whether cocaine use was assessed prior to each TMS session. Future studies that clarify this parameter would benefit the field of TMS research.

**Table 2:** Studies of TMS in CUD not requiring abstinence prior to TMS delivery.

Study	Study design, TMS parameters, assessment of cocaine use
Politi et al., 2008 (68)	Open label (n=36); L DLPFC (10 daily sessions: 15 Hz at 100% MT, presumed fig 8). Detoxification treatment prior to TMS, craving measured but not cocaine use. No serious adverse events.
Bolloni et al., 2016 (69)	Double blind randomized (n=10 active; n=8 sham); bilateral PFC (3 sessions/week: 10 Hz at 100% resting MT over 4 weeks; H1 coil). Cocaine hair analysis at baseline and 1,3,6 months. Four subjects were excluded due to high cocaine use at screening. No serious adverse events (but headache reported).
Rapinesi et al., 2016 (84)	Open label (n=7); L (preferential) DLPFC (12 sessions: 15 Hz at 100% MT over 1 month; H1 coil). Craving but not cocaine use obtained. No serious adverse events.
Terraneo et al., 2016 (70) *	Open label randomized (n=16 active; n=16 pharmacological control); L DLPFC (1 session/day:15 Hz at 90% resting MT for 5 days then 1 session/week x 3 weeks, fig 8). UDS obtained at each TMS visit, no report of sessions being held for a positive result. No serious adverse events.
Kearney-Ramos et al., 2018 (83)	Open label (n=49); frontal pole (TMS during brain scan, single pulses at 100% resting MT every 10–12 seconds on 1 day; fig 8). Cocaine use self-report obtained; whether TMS session held (or not) for recent use not specified. No serious adverse events.
Cardullo et al., 2019 (71)	Case series of CUD with gambling (n=7); L DLPFC (twice daily sessions for 5 days: 15 Hz at 100% MT followed by twice daily sessions once/week for 8 weeks, fig 8). UDS and self-report obtained as clinical outcome; whether TMS session held (or not) for recent use not specified. No serious adverse events.
Pettorruso et al., 2019 (72)	Open label (n=20); L DLPFC (5 sessions/week: 15 Hz at 100% resting MT for 2 weeks, then TMS twice/day for 2 weeks, fig 8). UDS and self-report obtained; whether TMS session held (or not) for recent use not specified. No serious adverse events.
Sanna et al., 2019 (82)	Open label randomized (n=25 iTBS; n=22–15 Hz TMS); bilateral PFC (iTBS: 5 Hz at 80% MT; 20 sessions in 4 weeks; 15 Hz TMS: 15 Hz at resting 100% MT; 20 sessions in 4 weeks; H4). UDS obtained at baseline and twice weekly during TMS sessions; whether TMS session held (or not) for recent use not specified. No serious adverse events.
Steele et al., 2019 (74) *	Open label (n=19); L DLPFC (3 iTBS sessions per day: 50 Hz at 100% resting MT for 10 days over 2 weeks). UDS obtained, 73% of TMS sessions performed with positive UDS, fig 8). Neurological event of uncertain etiology (right-hand supination/pronation at the wrist) 10–15 min following iTBS.
Gomez Perez et al., 2020 (81)	Retrospective observational study (n=87); L DLPFC (2 sessions/day at 15 Hz with 100% resting MT for 5 days; then 2 sessions/week for 12 weeks, butterfly). UDS, self-report, and reports from significant others were obtained at each TMS visit; whether TMS session held (or not) for recent use not specified. No serious adverse events.
Madeo et al., 2020 (75) *	Retrospective observational study (n=284); L DLPFC (2 x day sessions:15 Hz at 100% MT for 5 days, then 2 x day sessions once weekly x 12 weeks, fig 8). UDS, self-report, and reports from significant others at each TMS visit. No report of sessions held due to use. Seizure in 1 participant - not close to the recent session. Additional events: headache, dizziness, anxiety, hypomania, nausea, irritability, dental/scalp discomfort.
Cardullo et al., 2021 (76)	Retrospective, open label ADHD/CUD (n=22) and CUD-only (n=208); L DLPFC (twice/day of 15 Hz at 100% MT for 5 days then twice daily sessions once a week for 12 weeks, fig 8). UDS, self-report, or reports from significant others obtained at each visit; whether TMS session held (or not) for recent use not specified. No serious adverse events.
Garza-Villarreal et al., 2021 (77)	Double-blind, randomized (n=24 active; n=20 sham); L DLPFC (twice daily sessions of 5Hz at 100% MT for 10 days, then two sessions per week for up to 6 months, fig 8). UDS obtained at baseline, 2 weeks, 3 months, and 6 months; whether TMS session held (or not) for recent use not specified. No serious adverse events.
Lolli et al., 2021 (78)	Double-blind, randomized (n=32 active; n=30 sham); L DLPFC (15 sessions: 15 Hz at 100% resting MT over 3 weeks, fig 8). UDS obtained at baseline and twice weekly; whether TMS session held (or not) for recent use not specified. One participant in sham group experienced mild and transient paresthesia. No serious adverse events.
Martinotti et al., 2022 (85) *	Double-blind, randomized (n=42 active; n=38 sham) L DLPFC (20 sessions over 4 weeks:15 Hz at 100% resting MT for 5 days then 2 sessions/week for 12 weeks, butterfly). UDS and self-report obtained at each TMS visit. No reports of TMS being held for recent use. No serious adverse events in treatment phase, 1 participant in maintenance phase had seizure-like activity after cocaine use.
Sanna et al., 2022 (73)	Retrospective analysis (n=89); bilateral PFC and insula (20 sessions over 4 weeks at 50 Hz with 100% resting MT, H4). UDS obtained twice weekly, self-report weekly; whether TMS session held (or not) for recent use not specified. No serious adverse events (headache, dizziness, sleepiness, and insomnia reported).
Angeles-Valdez et al., 2024 (79)	Double-blind randomized clinical trial (n=30 active; n=24 sham); L DLPFC (Acute phase: 2 sessions/day at 5 Hz 100% MT for 10 days; open label maintenance phase: 2 sessions per week 5 Hz,100% MT, fig 8). UDS was performed prior to fMRI scans; whether TMS session held (or not) for recent use not specified. No serious adverse events.
Cardullo et al., 2024 (80)	Open label (n=40); L DLPFC (2 sessions/day at 15 Hz with 100% resting MT for 5 days, fig 8) UDS, self-report, and reports from significant others were obtained; whether TMS session held (or not) for recent use not specified. No serious adverse events
De Rossi et al., 2026 (86)	Open label (n=33); L DLPFC. (2 sessions/day at 15 Hz with 100% resting MT for 5 days; then 2 sessions/week for 3 months; then as needed sessions depending on clinical need, butterfly). UDS, self-report, and reports from significant others were obtained; whether TMS session held (or not) for recent use not specified. No serious adverse events.
Casula et al., 2025 (87)	Randomized clinical trial (n=19 active; n=23 sham); L DLPFC (1session/day for 2 weeks at 10 Hz with 90% MT, fig 8). UDS not obtained; whether TMS session held (or not) for recent use not specified. No serious adverse events.
Gomez Perez et al., 2025 (88)	Retrospective observational study (n=1011); L DLPFC (2 sessions/day at 15 Hz with 100% resting MT for 5 days; then 2 sessions/week for 12 weeks; then discretion of the treatment team could add iTBS to L DLPFC targeting anti-cocaine craving or low-frequent TMS over the presupplementary motor area for perseverative cocaine use; butterfly). UDS and self-report were obtained; whether TMS session held (or not) for recent use not specified. Reports of headache, scalp discomfort, along with 2 cases of hypomania, 2 cases of psychosis, 1 migraine, and 1 left upper limb paresthesia that resolved after stoppage of TMS.

CUD, cocaine use disorder; DLPFC, dorsolateral prefrontal cortex; iTBS, intermittent theta-burst stimulation; fig 8, figure-of-8-coil; MT, motor threshold; UDS, urine drug screen. Some studies obtained measures of cocaine use, such as urine drug screen (UDS), hair analysis, self-report, reports from significant others, or known detoxification. Of these, 5 specified whether cocaine use was obtained prior to the TMS sessions – as indicated with *.

Among the studies of TMS in CUD, we found two reports of a seizure (75, 85). Madeo et al. (75) performed an observational study (n = 248) and reported that one participant experienced a seizure following cocaine use. The authors reported that 66 days had passed since the first TMS session, but the precise time between the most recent TMS session and the seizure was not published (although the authors stated that TMS had not recently been delivered). Martinotti et al. (85) performed a randomized, sham-controlled study (n = 80) and reported a seizure-like episode in one participant in the active TMS group. The event occurred following intravenous cocaine use, but the proximity to the TMS session was not reported. Moreover, both studies were limited in their description of seizure-like activity and appeared to rely on self-report (75, 85).

Steele et al. (74) reported a transient neurological event of uncertain etiology in an open-label pilot study (n = 19) where a participant experienced rhythmic right-hand supination and pronation at the wrist. The event began 10–15 minutes following TMS and persisted for about 3 minutes (74, 89). The episode resolved within 1 hour, and no further TMS sessions were administered. Steele et al. (90) also reported a new-onset tremor in a participant with CUD who received sham intermittent theta burst stimulation.

Taken together, this data suggests that seizure is uncommon in studies using TMS in CUD. This finding is consistent with a recent meta-analysis, which reported a seizure prevalence of 2.32 per thousand participants receiving active TMS for individuals with CUD (91). However, clinical studies that describe the temporal association between cocaine use and the delivery of TMS, and the time between TMS and serious adverse events, such as seizure, would further demonstrate the safety of this intervention.

## CUD and motor threshold

Previous TMS studies have shown that participants with CUD have a higher motor threshold compared to controls (63, 66, 92, 93). Hanlon et al. (93) investigated the mechanism behind this using intracortical facilitation and functional MRI. The findings suggest that cocaine use is associated with reduced cortical facilitation, which may increase motor threshold and alter motor cortex BOLD signal, potentially mediated by glutamate and GABA signaling (93).

It remains unknown whether the elevated motor threshold seen in CUD varies with the amount of use or the duration of abstinence. Some TMS in CUD studies measured motor threshold prior to each session (59, 61, 74), which would address this potential variability. Alternatively, a recent study obtained a UDS prior to each TMS session, and the motor threshold was reassessed in the event of a newly positive result (NCT04907357). Either of these approaches would be expected to accommodate possible changes in cortical excitability.

## Discussion

Pharmacological treatment for CUD has been elusive, despite numerous clinical trials (3, 4). Additional treatments are needed, and research investigating TMS for CUD shows that this intervention reduces craving and cocaine use (6, 7, 94). Although pivotal clinical trials are still needed, there is a strong rationale for pursuing TMS as a therapy.

Given the potential benefit of TMS, there is a need to characterize the risk of seizure in CUD (95). To this end, we reviewed the research on cocaine exposure and seizure risk, which showed that patients requiring urgent medical care due to recent, high dose cocaine use were at risk for seizure. Outside of this scenario, there are no published reports of increased seizure rates among individuals with CUD. This suggests that CUD itself does not increase seizure risk – although this absence of documentation is not definitive.

**Figure 2:**
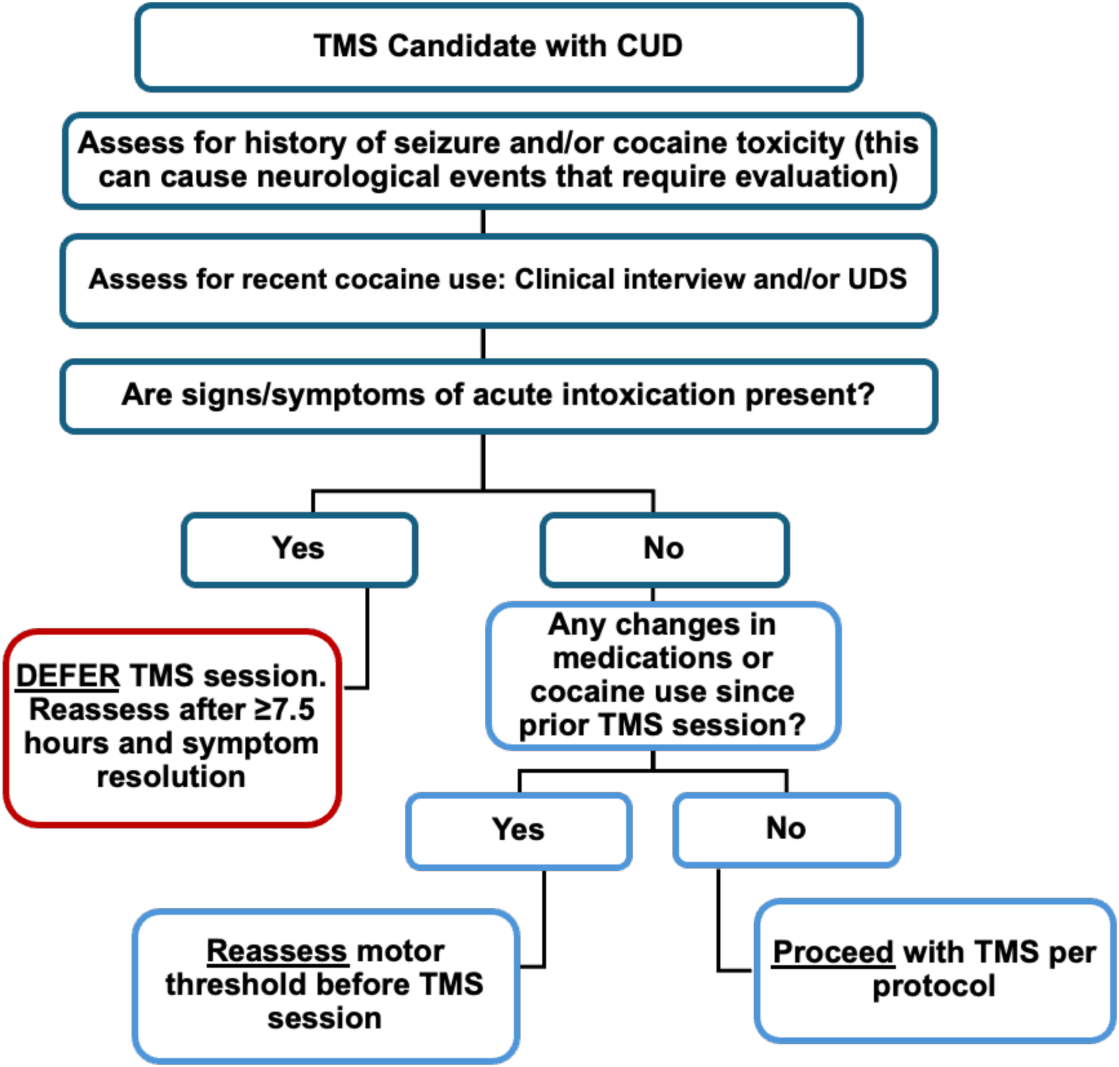
Decision tree for administering TMS in individuals with CUD.

With respect to TMS, the following steps, outlined in **Figure 2**, would be expected to mitigate seizure risk:

### An assessment of cocaine use prior to sessions

This can be done with a urine drug screen, although it would remain positive for 2–4 days, well after cocaine has cleared the plasma (33). An additional method is to establish a collaborative dialogue with individuals regarding their patterns of use, combined with observation for signs and symptoms of acute intoxication. In this approach, TMS sessions could be timed to avoid cocaine plasma levels above negligible amounts. The half-life of cocaine is about 90 minutes, meaning that plasma levels should be cleared by about 7.5 hours.

### An evaluation of cocaine toxicity

Individuals who use cocaine generally avoid toxic doses, but miscalculations can occur. A toxic dose (also called overdose) can include seizure in addition to other serious adverse events. An assessment of overdose should include both recent and past events, which may require a clinical evaluation. Additionally, psychoeducation on the risks associated with overdose, and the need for medical care, can serve as a form of harm reduction.

### A motor threshold assessment

Previous studies on TMS indicate that seizure risk is mitigated using a careful assessment of motor threshold, especially in the setting of medication changes (8, 9). Thus, checking motor threshold at each TMS session or rechecking when the individuals’ pattern of cocaine use changes would also be expected to reduce risk.

Overall, TMS has been investigated as a potential treatment for CUD, with promising results and few serious adverse events. Among these publications, there have been two reports of seizures, although both cases occurred following cocaine use – and not in temporal proximity to the TMS session (although the precise time frame was not reported). At the same time, it is notable that most studies did not clearly report whether recent cocaine use was assessed prior to the TMS sessions. This omission limits our ability to interpret the temporal relationship between recent cocaine use and TMS outcomes. Incorporating systematic assessment of recent cocaine use prior to TMS delivery in future studies would benefit the field and allow further documentation of the safety of TMS in CUD.

As with any treatment study, all participants, including CUD participants, should have a clinical evaluation that is specific to the population and study needs. The information provided here, while generally applicable in the design of future TMS studies, cannot replace clinical oversight and monitoring, which is important for research using TMS for any indication.

## Limitations

Our review of the literature has several limitations. As noted, there is substantial heterogeneity across studies in TMS protocols, approaches to assessing recent cocaine use, and methods for reporting seizure-like activity. These discrepancies hinder our ability to understand how TMS, cocaine use, and seizure events are related in terms of timing and sequence. In addition, a recent meta-analysis (91) that included several of the cocaine studies discussed here found a high risk of bias in these studies.

Furthermore, our publication did not review the efficacy of TMS for CUD, nor the occurrence of adverse events other than seizure. For these topics, we refer the reader to systematic reviews of TMS in SUD (6, 96) and substance use and to Blyth et al. (91), who reviewed the overall risk of adverse events in this patient population.

## References

[1] Kampman KM . The treatment of cocaine use disorder. Sci Adv. (2019) 5:eaax1532. doi: 10.1126/sciadv.aax1532, PMID: 31663022 PMC6795516

[2] Brandt L Chao T Comer SD Levin FR . How can we optimally channel therapeutic optimism to advance pharmacotherapy research on cocaine use disorder? Addiction. (2021) 116:715–7. doi: 10.1111/add.15423, PMID: 33538068

[3] Substance Abuse and Mental Health Services Administration. 2021 National Survey on Drug Use and Health (NSDUH): annual national report. Rockville (MD): Center for Behavioral Health Statistics and Quality (2023).

[4] Schwartz EK Wolkowicz NR Aquino JPD MacLean RR Sofuoglu M . Cocaine use disorder (CUD): current clinical perspectives. Subst Abus Rehabil. (2022) 13:25–46. doi: 10.2147/sar.s337338, PMID: 36093428 PMC9451050

[5] Goldstein RZ . Continuous theta burst stimulation of the medial prefrontal cortex reduces drug cue reactivity with a potential for improving outpatient treatment outcomes in cocaine use disorder. Biol Psychiatry: Cognit Neurosci Neuroimaging. (2025) 10:557–9. doi: 10.1016/j.bpsc.2025.04.007, PMID: 40483007 PMC13504516

[6] Amerio A Baccino C Breda GS Cortesi D Spiezio V Magnani L . Effects of transcranial magnetic stimulation on cocaine addiction: A systematic review of randomized controlled trials. Psychiatry Res. (2023) 329:115491. doi: 10.1016/j.psychres.2023.115491, PMID: 37783092

[7] Harmelech T Hanlon CA Tendler A . Transcranial magnetic stimulation as a tool to promote smoking cessation and decrease drug and alcohol use. Brain Sci. (2023) 13:1072. doi: 10.3390/brainsci13071072, PMID: 37509004 PMC10377606

[8] Stultz DJ Osburn S Burns T Pawlowska-Wajswol S Walton R . Transcranial magnetic stimulation (TMS) safety with respect to seizures: A literature review. Neuropsychiatr Dis Treat. (2020) 16:2989–3000. doi: 10.2147/ndt.s276635, PMID: 33324060 PMC7732158

[9] Tendler A Harmelech T Gersner R Roth Y . Seizures provoked by H-coils from 2010 to 2020. Brain Stimul. (2021) 14:66–8. doi: 10.1016/j.brs.2020.11.006, PMID: 33197655

[10] Lowenstein DH Massa SM Rowbotham MC Collins SD McKinney HE Simon RP . Acute neurologic and psychiatric complications associated with cocaine abuse. Am J Med. (1987) 83:841–6. doi: 10.1016/0002-9343(87)90640-1, PMID: 3674091

[11] Choy-Kwong M Lipton RB . Seizures inhospitalized cocaine users. Neurology. (1989) 39:425–5. doi: 10.1212/wnl.39.3.425, PMID: 2927655

[12] Derlet RW Albertson TE . Emergency department presentation of cocaine intoxication. Ann Emerg Med. (1989) 18:182–6. doi: 10.1016/s0196-0644(89)80111-8, PMID: 2916784

[13] Pascual-Leone A Dhuna A Altafullah I Anderson DC . Cocaine-induced seizures. Neurology. (1990) 40:404–4. doi: 10.1212/wnl.40.3_part_1.404, PMID: 2107459

[14] Brody SL Slovis CM Wrenn KD . Cocaine-related medical problems: Consecutive series of 233 patients. Am J Med. (1990) 88:325–31. doi: 10.1016/0002-9343(90)90484-u, PMID: 2327419

[15] Dhuna A Pascual-Leone A Langendorf F Anderson DC . Epileptogenic properties of cocaine in humans. Neurotoxicology. (1991) 12:621–6. doi: 10.1016/0161-813X(91)90005-3, PMID: 1745445

[16] Rich JA Singer DE . Cocaine-related symptoms in patients presenting to an urban emergency department. Ann Emerg Med. (1991) 20:616–21. doi: 10.1016/s0196-0644(05)82378-9, PMID: 2039099

[17] Anta GB Arenas MAR de HL Morales LR Work Group for the Study of Emergencies from Psychostimulants] . Emergency room admissions in cocaine users in Spanish hospitals: first evidences of acute complications related to crack use. Med Clin. (1998) 111:49–55. doi: 10.1016/0161-813X(91)90005-3 9706586

[18] Blaho K Logan B Winbery S Park L Schwilke E . Blood cocaine and metabolite concentrations, clinical findings, and outcome of patients presenting to an ED. Am J Emerg Med. (2000) 18:593–8. doi: 10.1053/ajem.2000.9282, PMID: 10999576

[19] Sanjurjo E Montori E Nogué S Sánchez M Munné P . Cocaine abuse attended in the emergency department: an emerging pathology. Med Clin. (2006) 126:616–9. doi: 10.1157/13087719, PMID: 16826639

[20] Sopeña B Rivera A Rodríguez-Domínguez M Rodríguez-Rodríguez M Argibay A Maure B . Complications related with cocaine abuse that required hospital admission. Rev Clin espanola. (2008) 208:12–7. doi: 10.1157/13115002, PMID: 18221656

[21] Ng SKC Brust JCM Hauser WA Susser M . Illicit drug use and the risk of new-onset seizures. Am J Epidemiol. (1990) 132:47–57. doi: 10.1093/oxfordjournals.aje.a115642, PMID: 2356813

[22] Glidden E Suen K Mustaquim D Vivolo-Kantor A Brent J Wax P . Characterization of nonfatal opioid, cocaine, methamphetamine, and polydrug exposure and clinical presentations reported to the toxicology investigators consortium core registry, january 2010–december 2021. J Med Toxicol. (2023) 19:180–9. doi: 10.1007/s13181-022-00924-0, PMID: 36650409 PMC10050626

[23] Darke S Duflou J Peacock A Chrzanowska A Farrell M Lappin J . Clinical characteristics of fatal cocaine toxicity in Australia, 2000–2021. Drug Alcohol Rev. (2023) 42:582–91. doi: 10.1111/dar.13581, PMID: 36423902

[24] Bodmer M Enzler F Liakoni E Bruggisser M Liechti ME . Acute cocaine-related health problems in patients presenting to an urban emergency department in Switzerland: a case series. BMC Res Notes. (2014) 7:173. doi: 10.1186/1756-0500-7-173, PMID: 24666782 PMC3987164

[25] Miró ÒChecktae Dargan PI Wood DM Dines AM Yates C Heyerdahl F . Epidemiology, clinical features and management of patients presenting to European emergency departments with acute cocaine toxicity: comparison between powder cocaine and crack cocaine cases. Clin Toxicol. (2019) 57:718–26. doi: 10.1080/15563650.2018.1549735, PMID: 30696283

[26] Miró Ò Galicia M Dargan PI Wood DM Dines AM Heyerdahl F . Age and sex related differences in clinical manifestations and severity of acute cocaine toxicity presentations to European emergency departments. Am J Emerg Med. (2025) 96:151–60. doi: 10.1016/j.ajem.2025.06.035, PMID: 40570490

[27] Sordo L Indave BI Degenhardt L Barrio G Kaye S Ruíz-Pérez I . A systematic review of evidence on the association between cocaine use and seizures. Drug Alcohol Depend. (2013) 133:795–804. doi: 10.1016/j.drugalcdep.2013.08.019, PMID: 24051062

[28] Hughto JMW Kelly PJA Vento SA Pletta DR Noh M Silcox J . Characterizing and responding to stimulant overdoses: Findings from a mixed methods study of people who use cocaine and other stimulants in New England. Drug Alcohol Depend. (2025) 266:112501. doi: 10.1016/j.drugalcdep.2024.112501, PMID: 39608288 PMC11685032

[29] Schwartz RH Luxenberg MG Hoffmann NG . Crack” use by american middle-class adolescent polydrug abusers. J Pediatr. (1991) 118:150–5. doi: 10.1016/s0022-3476(05)81871-2, PMID: 1986085

[30] Mansoor M McNeil R Fleming T Baker A Vakharia S Sue K . Characterizing stimulant overdose: A qualitative study on perceptions and experiences of “overamping. ” Int J Drug Polic. (2022) 102:103592. doi: 10.1016/j.drugpo.2022.103592, PMID: 35114520 PMC9381030

[31] Ferri CP Dunn J Gossop M Laranjeira R . Factors associated with adverse reactions to cocaine among a sample of long-term, high-dose users in São Paulo, Brazil. Addict Behav. (2004) 29:365–74. doi: 10.1016/j.addbeh.2003.08.029, PMID: 14732425

[32] Kaye S Darke S . Injecting and non-injecting cocaine use in Sydney, Australia: physical and psychological morbidity. Drug Alcohol Rev. (2004) 23:391–8. doi: 10.1080/09595230412331324518, PMID: 15763743

[33] Moeller KE Lee KC Kissack JC . Urine drug screening: practical guide for clinicians. Mayo Clin Proc. (2008) 83:66–76. doi: 10.4065/83.1.66, PMID: 18174009

[34] Gitto L Mir M Arunkumar P . Evaluating the reliability of dipstick drug screens on vitreous and postmortem blood as a triage modality in forensic pathology. Acad Forensic Pathol. (2023) 13:92–100. doi: 10.1177/19253621231190415, PMID: 38298544 PMC10825466

[35] Puet BL Claussen K Hild C Heltsley R Schwope DM . Presence of parent cocaine in the absence of benzoylecgonine in urine†. J Anal Toxicol. (2018) 42:512–7. doi: 10.1093/jat/bky057, PMID: 30371845

[36] Huestis MA Darwin WD Shimomura E Lalani SA Trinidad DV Jenkins AJ . Cocaine and metabolites urinary excretion after controlled smoked administration*. J Anal Toxicol. (2007) 31:462–8. doi: 10.1093/jat/31.8.462, PMID: 17988460 PMC3128807

[37] Chow MJ Ambre JJ Ruo TI Atkinson AJ Bowsher DJ Fischman MW . Kinetics of cocaine distribution, elimination, and chronotropic effects. Clin Pharmacol Ther. (1985) 38:318–24. doi: 10.1038/clpt.1985.179, PMID: 4028628

[38] Isenschmid DS Fischman MW Foltin RW Caplan YH . Concentration of cocaine and metabolites in plasma of humans following intravenous administration and smoking of cocaine. J Anal Toxicol. (1992) 16:311–4. doi: 10.1093/jat/16.5.311, PMID: 1294836

[39] Wilkinson P Dyke CV Jatlow P Barash P Byck R . Intranasal and oral cocaine kinetics. Clin Pharmacol Ther. (1980) 27:386–94. doi: 10.1038/clpt.1980.52, PMID: 7357795

[40] Foltin RW Fischman MW . Smoked and intravenous cocaine in humans: acute tolerance, cardiovascular and subjective effects. J Pharmacol Exp Ther. (1991) 257:247–61. doi: 10.1016/S0022-3565(25)24787-5 2019989

[41] Ward AS Haney M Fischman MW Foltin RW . Binge cocaine self-administration in humans: intravenous cocaine. Psychopharmacology. (1997) 132:375–81. doi: 10.1007/s002130050358, PMID: 9298515

[42] Ward AS Haney M Fischman MW Foltin RW . Binge cocaine self-administration by humans: smoked cocaine. Behav Pharmacol. (1997) 8:736–44. doi: 10.1097/00008877-199712000-00009, PMID: 9832960

[43] Foltin RW Ward AS Haney M Hart CL Collins ED . The effects of escalating doses of smoked cocaine in humans. Drug Alcohol Depend. (2003) 70:149–57. doi: 10.1016/s0376-8716(02)00343-5, PMID: 12732408

[44] Dudish-Poulsen S Hatsukami DK . Acute abstinence effects following smoked cocaine administration in humans. Exp Clin Psychopharmacol. (2000) 8:472–82. doi: 10.1037/1064-1297.8.4.472, PMID: 11127419

[45] Evans SM Cone EJ Henningfield JE . Arterial and venous cocaine plasma concentrations in humans: relationship to route of administration, cardiovascular effects and subjective effects. J Pharmacol Exp Ther. (1996) 279:1345–56. doi: 10.1016/S0022-3565(25)21295-2, PMID: 8968359

[46] Walsh SL Donny EC Nuzzo PA Umbricht A Bigelow GE . Cocaine abuse versus cocaine dependence: Cocaine self-administration and pharmacodynamic response in the human laboratory. Drug Alcohol Depend. (2010) 106:28–37. doi: 10.1016/j.drugalcdep.2009.07.011, PMID: 19717246 PMC2815181

[47] Javaid JI Fischman MW Schuster CR Dekirmenjian H Davis JM . Cocaine plasma concentration: relation to physiological and subjective effects in humans. Science. (1978) 202:227–8. doi: 10.1126/science.694530, PMID: 694530

[48] Foltin RW Haney M . Intranasal cocaine in humans: acute tolerance, cardiovascular and subjective effects. Pharmacol Biochem Behav. (2004) 78:93–101. doi: 10.1016/j.pbb.2004.02.018, PMID: 15159138

[49] Stoops WW Vansickel AR Lile JA Rush CR . Acute d-amphetamine pretreatment does not alter stimulant self-administration in humans. Pharmacol Biochem Behav. (2007) 87:20–9. doi: 10.1016/j.pbb.2007.03.016, PMID: 17490738 PMC2045695

[50] Donny EC Bigelow GE Walsh SL . Assessing the initiation of cocaine self-administration in humans during abstinence: effects of dose, alternative reinforcement, and priming. Psychopharmacology. (2004) 172:316–23. doi: 10.1007/s00213-003-1655-z, PMID: 14647955

[51] Foltin RW Fischman MW . Effects of “binge” use of intravenous cocaine in methadone-maintained individuals. Addiction. (1998) 93:825–36. doi: 10.1046/j.1360-0443.1998.9368254.x, PMID: 9744118

[52] Evans SM Haney M Fischman MW Foltin RW . Limited sex differences in response to “Binge” Smoked cocaine use in humans. Neuropsychopharmacology. (1999) 21:445–54. doi: 10.1016/s0893-133x(98)00120-1, PMID: 10457542

[53] Epstein DH Silverman K Henningfield JE Preston KL . Low-dose oral cocaine in humans: acquisition of discrimination and time-course of effects. Behav Pharmacol. (1999) 10:531–42. doi: 10.1097/00008877-199909000-00011, PMID: 10780259

[54] Richards JR Le JK . Cocaine toxicity. In: StatPearls. [Internet] Treasure Island (FL): StatPearls Publishing. (2025). Available online at: https://www.ncbi.nlm.nih.gov/books/NBK430976/. 28613695

[55] Treadwell SD Robinson TG . Cocaine use and stroke. Postgrad Med J. (2007) 83:389. doi: 10.1136/pgmj.2006.055970, PMID: 17551070 PMC2600058

[56] Rendon LF Malta S Leung J Badenes R Nozari A Bilotta F . Cocaine and ischemic or hemorrhagic stroke: A systematic review and meta-analysis of clinical evidence. J Clin Med. (2023) 12:5207. doi: 10.3390/jcm12165207, PMID: 37629248 PMC10455873

[57] Camprodon JA Martínez-Raga J Alonso-Alonso M Shih M-C Pascual-Leone A . One session of high frequency repetitive transcranial magnetic stimulation (rTMS) to the right prefrontal cortex transiently reduces cocaine craving. Drug Alcohol Depend. (2007) 86:91–4. doi: 10.1016/j.drugalcdep.2006.06.002, PMID: 16971058

[58] Hanlon CA Dowdle LT Austelle CW DeVries W Mithoefer O Badran BW . What goes up, can come down: Novel brain stimulation paradigms may attenuate craving and craving-related neural circuitry in substance dependent individuals. Brain Res. (2015) 1628:199–209. doi: 10.1016/j.brainres.2015.02.053, PMID: 25770818 PMC4899830

[59] Hanlon CA Dowdle LT Correia B Mithoefer O Kearney-Ramos T Lench D . Left frontal pole theta burst stimulation decreases orbitofrontal and insula activity in cocaine users and alcohol users. Drug Alcohol Depend. (2017) 178:310–7. doi: 10.1016/j.drugalcdep.2017.03.039, PMID: 28686990 PMC5896018

[60] Kearney-Ramos TE Dowdle LT Mithoefer OJ Devries W George MS Hanlon CA . State-dependent effects of ventromedial prefrontal cortex continuous thetaburst stimulation on cocaine cue reactivity in chronic cocaine users. Front Psychiatry. (2019) 10:317. doi: 10.3389/fpsyt.2019.00317, PMID: 31133897 PMC6517551

[61] Martinez D Urban N Grassetti A Chang D Hu M-C Zangen A . Transcranial magnetic stimulation of medial prefrontal and cingulate cortices reduces cocaine self-administration: A pilot study. Front Psychiatry. (2018) 9:80. doi: 10.3389/fpsyt.2018.00080, PMID: 29615935 PMC5864905

[62] Kearney-Ramos TE Dowdle LT Lench DH Mithoefer OJ Devries WH George MS . Transdiagnostic effects of ventromedial prefrontal cortex transcranial magnetic stimulation on cue reactivity. Biol Psychiatry: Cognit Neurosci Neuroimaging. (2018) 3:599–609. doi: 10.1016/j.bpsc.2018.03.016, PMID: 29776789 PMC6641556

[63] Boutros NN Lisanby SH Tokuno H Torello MW Campbell D Berman R . Elevated motor threshold in drug-free, cocaine-dependent patients assessed with transcranial magnetic stimulation. Biol Psychiatry. (2001) 49:369–73. doi: 10.1016/s0006-3223(00)00948-3, PMID: 11239908

[64] Boutros NN Lisanby SH McClain-Furmanski D Oliwa G Gooding D Kosten TR . Cortical excitability in cocaine-dependent patients: a replication and extension of TMS findings. J Psychiatr Res. (2005) 39:295–302. doi: 10.1016/j.jpsychires.2004.07.002, PMID: 15725428

[65] Gjini K Qazi A Greenwald MK Sandhu R Gooding DC Boutros NN . Relationships of behavioral measures of frontal lobe dysfunction with underlying electrophysiology in cocaine-dependent patients. Am J Addict. (2014) 23:265–71. doi: 10.1111/j.1521-0391.2014.12095.x, PMID: 24724884 PMC3986823

[66] Sundaresan K Ziemann U Stanley J Boutros N . Cortical inhibition and excitation in abstinent cocaine-dependent patients&colon; a transcranial magnetic stimulation study. NeuroReport. (2007) 18:289–92. doi: 10.1097/wnr.0b013e3280143cf0, PMID: 17314673

[67] McCalley DM Kinney KR Kaur N Wolf JP Contreras IE Smith JP . A randomized controlled trial of medial prefrontal cortex theta burst stimulation for cocaine use disorder: A three-month feasibility and brain target engagement study. Biol Psychiatry: Cognit Neurosci Neuroimaging. (2025) 10:616–25. doi: 10.1016/j.bpsc.2024.11.022, PMID: 39667495 PMC13151909

[68] Politi E Fauci E Santoro A Smeraldi E . Daily sessions of transcranial magnetic stimulation to the left prefrontal cortex gradually reduce cocaine craving. Am J Addict. (2008) 17:345–6. doi: 10.1080/10550490802139283, PMID: 18612892

[69] Bolloni C Panella R Pedetti M Frascella AG Gambelunghe C Piccoli T . Bilateral transcranial magnetic stimulation of the prefrontal cortex reduces cocaine intake: A pilot study. Front Psychiatry. (2016) 7:133. doi: 10.3389/fpsyt.2016.00133, PMID: 27551268 PMC4976094

[70] Terraneo A Leggio L Saladini M Ermani M Bonci A Gallimberti L . Transcranial magnetic stimulation of dorsolateral prefrontal cortex reduces cocaine use: A pilot study. Eur Neuropsychopharmacol. (2016) 26:37–44. doi: 10.1016/j.euroneuro.2015.11.011, PMID: 26655188 PMC9379076

[71] Cardullo S Perez LJG Marconi L Terraneo A Gallimberti L Bonci A . Clinical improvements in comorbid gambling/cocaine use disorder (GD/CUD) patients undergoing repetitive transcranial magnetic stimulation (rTMS). J Clin Med. (2019) 8:768. doi: 10.3390/jcm8060768, PMID: 31151221 PMC6616893

[72] Pettorruso M Martinotti G Santacroce R Montemitro C Fanella F Giannantonio M . rTMS reduces psychopathological burden and cocaine consumption in treatment-seeking subjects with cocaine use disorder: an open label, feasibility study. Front Psychiatry. (2019) 10:621. doi: 10.3389/fpsyt.2019.00621, PMID: 31543838 PMC6739618

[73] Sanna A Bini V Badas P Corona G Sanna G Marcasciano L . Role of maintenance treatment on long-term efficacy of bilateral iTBS of the prefrontal cortex in treatment-seeking cocaine addicts: A retrospective analysis. Front Psychiatry. (2022) 13:1013569. doi: 10.3389/fpsyt.2022.1013569, PMID: 36424992 PMC9679214

[74] Steele VR Maxwell AM Ross TJ Stein EA Salmeron BJ . Accelerated intermittent theta-burst stimulation as a treatment for cocaine use disorder: A proof-of-concept study. Front Neurosci. (2019) 13:1147. doi: 10.3389/fnins.2019.01147, PMID: 31736689 PMC6831547

[75] Madeo G Terraneo A Cardullo S Pérez LJG Cellini N Sarlo M . Long-term outcome of repetitive transcranial magnetic stimulation in a large cohort of patients with cocaine-use disorder: an observational study. Front Psychiatry. (2020) 11:158. doi: 10.3389/fpsyt.2020.00158, PMID: 32180745 PMC7059304

[76] Cardullo S Pérez LJG Cuppone D Sarlo M Cellini N Terraneo A . A retrospective comparative study in patients with cocaine use disorder comorbid with attention deficit hyperactivity disorder undergoing an rTMS protocol treatment. Front Psychiatry. (2021) 12:659527. doi: 10.3389/fpsyt.2021.659527, PMID: 33841218 PMC8026860

[77] Garza-Villarreal EA Alcala-Lozano R Fernandez-Lozano S Morelos-Santana E Dávalos A Villicaña V . Clinical and functional connectivity outcomes of 5-hz repetitive transcranial magnetic stimulation as an add-on treatment in cocaine use disorder: A double-blind randomized controlled trial. Biol Psychiatry: Cognit Neurosci Neuroimaging. (2021) 6:745–57. doi: 10.1016/j.bpsc.2021.01.003, PMID: 33508499

[78] Lolli F Salimova M Scarpino M Lanzo G Cossu C Bastianelli M . A randomised, double-blind, sham-controlled study of left prefrontal cortex 15 Hz repetitive transcranial magnetic stimulation in cocaine consumption and craving. PloS One. (2021) 16:e0259860. doi: 10.1371/journal.pone.0259860, PMID: 34784373 PMC8594832

[79] Angeles-Valdez D Rasgado-Toledo J Villicaña V Davalos-Guzman A Almanza C Fajardo-Valdez A . The Mexican dataset of a repetitive transcranial magnetic stimulation clinical trial on cocaine use disorder patients: SUDMEX TMS. Sci Data. (2024) 11:408. doi: 10.1038/s41597-024-03242-y, PMID: 38649689 PMC11035677

[80] Cardullo S Pérez LJG Terraneo A Gallimberti L Mioni G . Time perception in stimulant-dependent participants undergoing repetitive transcranial magnetic stimulation. Behav Brain Res. (2024) 460:114816. doi: 10.1016/j.bbr.2023.114816, PMID: 38122902

[81] Pérez LJG Cardullo S Cellini N Sarlo M Monteanni T Bonci A . Sleep quality improves during treatment with repetitive transcranial magnetic stimulation (rTMS) in patients with cocaine use disorder: a retrospective observational study. BMC Psychiatry. (2020) 20:153. doi: 10.1186/s12888-020-02568-2, PMID: 32252720 PMC7137315

[82] Sanna A Fattore L Badas P Corona G Cocco V Diana M . Intermittent theta burst stimulation of the prefrontal cortex in cocaine use disorder: A pilot study. Front Neurosci. (2019) 13:765. doi: 10.3389/fnins.2019.00765, PMID: 31402851 PMC6670008

[83] Kearney-Ramos TE Lench DH Hoffman M Correia B Dowdle LT Hanlon CA . Gray and white matter integrity influence TMS signal propagation: a multimodal evaluation in cocaine-dependent individuals. Sci Rep. (2018) 8:3253. doi: 10.1038/s41598-018-21634-0, PMID: 29459743 PMC5818658

[84] Rapinesi C Casale AD Pietro SD Ferri VR Piacentino D Sani G . Add-on high frequency deep transcranial magnetic stimulation (dTMS) to bilateral prefrontal cortex reduces cocaine craving in patients with cocaine use disorder. Neurosci Lett. (2016) 629:43–7. doi: 10.1016/j.neulet.2016.06.049, PMID: 27365134

[85] Martinotti G Pettorruso M Montemitro C Spagnolo PA Martellucci CA Carlo FD . Repetitive transcranial magnetic stimulation in treatment-seeking subjects with cocaine use disorder: A randomized, double-blind, sham-controlled trial. Prog Neuro-Psychopharmacol Biol Psychiatry. (2022) 116:110513. doi: 10.1016/j.pnpbp.2022.110513, PMID: 35074451

[86] Rossi ED Pérez LJG Cardullo S Carbone GA Zaffaina GC Farina B . Increased delta connectivity within brain networks as a biomarker of adherence to a rTMS-based treatment program in a sample of cocaine use disorder patients. J Stud Alcohol Drugs. (2026) 87:75–84. doi: 10.15288/jsad.24-00441, PMID: 40410942

[87] Casula EP Chieco F Papaioannou MM Frizzarin F Rocchi L Camporese A . Trend-analysis reveals real and placebo rtms effects on addiction craving: a case-control observational study. Front Psychiatry. (2025) 15:1441815. doi: 10.3389/fpsyt.2024.1441815, PMID: 40190483 PMC11969045

[88] Pérez LJG Cardullo S Zaffaina GC Cuppone D Chindamo S Zattin A . Prognostic factors of a multidisciplinary rtms-based treatment for cocaine use disorder: A large cohort study. J Subst Addict Treat. (2025) 174:209706. doi: 10.1016/j.josat.2025.209706, PMID: 40286859

[89] Steele VR Maxwell AM Ross TJ Moussawi K Abulseoud OO Stein EA . Report of transient events in a cocaine-dependent volunteer who received iTBS. Brain Stimul. (2018) 11:631–3. doi: 10.1016/j.brs.2018.01.004, PMID: 29429954

[90] Steele VR Rotenberg A Philip NS Hallett M Stein EA Salmeron BJ . Case report: Tremor in the placebo condition of a blinded clinical trial of intermittent theta-burst stimulation for cocaine use disorder. Front Psychiatry. (2024) 15:1391771. doi: 10.3389/fpsyt.2024.1391771, PMID: 39045554 PMC11263940

[91] Blyth SH Haq R Menon S King B Quesada GT Borodge D . Evidence for safety and tolerability of transcranial magnetic stimulation for substance use disorders. Brain Stimul. (2025) 18:2043–9. doi: 10.1016/j.brs.2025.11.002, PMID: 41197767 PMC12910563

[92] Gjini K Ziemann U Napier TC Boutros N . Dysbalance of cortical inhibition and excitation in abstinent cocaine-dependent patients. J Psychiatr Res. (2012) 46:248–55. doi: 10.1016/j.jpsychires.2011.10.006, PMID: 22036187 PMC3264814

[93] Hanlon CA DeVries W Dowdle LT West JA Siekman B Li X . A comprehensive study of sensorimotor cortex excitability in chronic cocaine users: Integrating TMS and functional MRI data. Drug Alcohol Depend. (2015) 157:28–35. doi: 10.1016/j.drugalcdep.2015.07.1196, PMID: 26541870 PMC4899825

[94] Steele VR Maxwell AM . Treating cocaine and opioid use disorder with transcranial magnetic stimulation: A path forward. Pharmacol Biochem Behav. (2021) 209:173240. doi: 10.1016/j.pbb.2021.173240, PMID: 34298030 PMC8445657

[95] Steele VR Addicott MA Addolorato G Baker T Biernacki K Bonci A . Toward inclusive, evidence-based rTMS care for patients with co-occurring substance use disorders. Am J Psychiatry. (2025) 182:1095–6. doi: 10.1176/appi.ajp.20250407, PMID: 41320823

[96] Mehta DD Praecht A Ward HB Sanches M Sorkhou M Tang VM . A systematic review and meta-analysis of neuromodulation therapies for substance use disorders. Neuropsychopharmacology. (2024) 49:649–80. doi: 10.1038/s41386-023-01776-0, PMID: 38086901 PMC10876556

